# The Herbst Methods

**Published:** 1886-10

**Authors:** 


### THE HERBST METHODS.

We are in receipt of a number of letters asking our opinion concerning the Herbst system of filling teeth, and whether, in our opinion, his methods will be generally adopted in this country. Some of the questions are exceedingly difficult ones to answer, but we have no hesitation in giving our impressions.

There is a great deal of good in the Herbst method. Probably no one who has ever visited us from abroad has produced so deep an impression upon professional minds, or has seen such immediate practical results flow from his teachings, as has Wilhelm Herbst. As we said in the August number, this is largely due to certain peculiar circumstances, and to the genuine enthusiasm, the unselfish devotion, as well as to the undoubted genius of Herbst himself. The way, also, had been carefully prepared for him by one in whom the profession of America has great confidence—Dr. Bödecker—who, himself thoroughly convinced of the merits of the Herbst system, in entire disinterestedness devoted his time and money to what he felt was the service, not so much of Dr. Herbst, as of the profession of America. It should be understood that the principal part of the expense of the whole tour has been borne by Dr. Bödecker, and American dentists owe him a debt which they can never repay.

But admitting all this, had the ideas of Herbst been without any practical value, they could not have produced upon professional minds here so deep an impression as they have. There is no question concerning their great utility, and in our mind there is little doubt that Dr. Herbst's visit will permanently modify American dental practice. Not that we are of the opinion that his ways will supersede ours, or that the rotary burnisher will displace the mallet. It would be nonsense to claim this. But the method of condensing gold is not all there is in Herbst's system. There are numberless little inventions and ideas which are peculiar to him, and which form a part of his gift to the dental profession. And a gift it is, for we should not forget that Herbst has never taken out a patent, nor sought in any way to control his devices.

The American dentist who did not witness an operation at the hands of Dr. Herbst has missed one of the opportunities of a lifetime. It is almost impossible to entirely comprehend his methods without witnessing them. He is original in all his ways, and his expedients are endless. Yet from the accounts of his clinics, as reported in the INDEPENDENT PRACTITIONER, a very clear idea of his manipulation of gold may, by careful study, be obtained.

Our advice to every operator is, that he carefully experiment in the Herbst system. Not with the expectation of abandoning the mallet, or any other established system, but that he may have command of a method which presents very many advantages, and which is peculiarly adapted to exigencies which often arise; a method by which results that are often desirable can more easily and perfectly be secured than in any other way, the study of which will certainly tend to give broader views of the possibilities of operative practice, and a knowledge of which will make better operators in the old established way, and assist to a more thorough comprehension of the underlying principles of operative dentistry.

